# Focal impulse and rotor modulation of atrial rotors during atrial fibrillation leads to organization of left atrial activation as reflected by waveform morphology recurrence quantification analysis and organizational index

**DOI:** 10.1002/joa3.12311

**Published:** 2020-02-24

**Authors:** Peter R. Liu, Daniel J. Friedman, Adam S. Barnett, Kevin P. Jackson, James P. Daubert, Jonathan P. Piccini

**Affiliations:** ^1^ Department of Medicine Johns Hopkins Hospital Baltimore MD USA; ^2^ Duke Center for Atrial Fibrillation Duke Clinical Research Institute Duke University Medical Center Durham NC USA

**Keywords:** atrial fibrillation, cardiac mapping, catheter ablation, electrogram analysis, rotor ablation

## Abstract

**Background:**

Focal impulse and rotor modulation (FIRM) can cause slowing, organization, and occasionally termination of atrial fibrillation (AF), although results have been mixed. To further characterize changes in AF during rotor ablation, we quantified morphologic and temporal activation changes following FIRM.

**Methods:**

In patients undergoing FIRM ablation for AF, we retrospectively analyzed coronary sinus bipolar EGMs before and after rotor ablation, including EGM activation frequency and regularity, dominant frequency (DF), and organizational index (OI). Changes in EGM waveform morphology were determined with recurrence quantification analysis (RQA) consisting of recurrence rate (RR), determinism (DET), laminarity (LAM), average diagonal line length (L), and trapping time (TT) using Wilcoxon signed‐rank testing.

**Results:**

Overall, 36 rotors from 21 patients undergoing FIRM ablation were analyzed. All morphology RQA parameters demonstrated significant organization of atrial activation after rotor ablation (RR *P* = .03, DET *P* = .005, LAM *P* = .03, L *P* = .005, TT *P* = .009). The organizational index also showed a significant increase after rotor ablation (*P* = .01), and the change in OI correlated with changes in all morphology parameters. Of the rotors, 14/36 (39%) rotors showed organizational changes in all morphology parameters and OI, and an additional 5 rotors (19/36, 53%) showed organizational changes in 4 of 5 morphology parameters and OI.

**Conclusions:**

Coronary sinus EGM waveform morphologies and activation patterns are significantly altered after FIRM ablation even when there is no fibrillatory slowing. RQA morphology analysis and organizational index may impart important information regarding underlying AF organization and may be useful in quantifying the acute response to ablation.

## Graphical abstract

We quantified changes in the morphologic and temporal activation patterns in left atrial electrograms before and after focal source/rotor ablation using FIRM. In our analysis, we found that coronary sinus electrogram waveform morphologies and activation patterns are significantly altered after FIRM ablation even when no fibrillatory rate slowing is clinically evident.

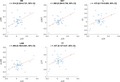

## 1 INTRODUCTION

Identification and ablation of extrapulmonary drivers of atrial fibrillation (AF) may improve maintenance of sinus rhythm after catheter ablation.^1^ Over the past several years, focal impulse and rotor modulation (FIRM) has emerged as an adjunctive ablation technique to sites of organized rotational activity during AF ablation. However, results following FIRM have been highly variable with anywhere between 17% and 80% freedom from AF at 1 year.^2^, ^3^, ^4^, ^5^ Numerous studies have observed acute procedural changes in atrial rhythm during FIRM ablation, such as a 10% or greater increase in the mean AF cycle length, conversion to a more regular atrial tachycardia, or termination of AF with restoration of sinus rhythm. However, these outcomes are also highly variable, ranging anywhere from 40% to 100%^4^, ^5^, ^6^, ^7^ depending upon the endpoint of interest. The variability of these results has called into question the methodology, reproducibility, and efficacy of FIRM to alter AF organization in the short term, and to prevent recurrent AF in the long term. A better understanding and description of the effects of FIRM ablation on AF organization would help provide insight into the mechanism and efficacy of FIRM while improving our ability to guide FIRM ablation during the ablation procedure.

Frequency domain spectral analysis can be used to quantify the dominant frequency (DF) of atrial activation, as well as its temporal stability via the organizational index (OI). These measures have been associated with organization and termination of AF following antiarrhythmic therapy and step‐wise catheter ablation.^8^, ^9^, ^10^, ^11^ While frequency domain analysis primarily describes temporal patterns of atrial activation, recurrence quantification analysis (RQA) techniques describe both morphologic^12^, ^13^, ^14^, ^15^ and temporal^16^, ^17^ stability of AF on the surface electrocardiogram, intracardiac EGMs, and epicardial EGMs. Analyses of intracardiac EGMs suggest that RQA may characterize underlying AF organization^15^ while identifying local areas that may be drivers of AF.^13^ Furthermore, RQA parameters have been shown to discriminate among paroxysmal, persistent, and induced AF,^12^ predict spontaneous termination of AF,^18^ and predict long‐term catheter ablation outcomes.^17^

The goal of this analysis was to test the hypothesis that FIRM ablation results in quantifiable temporal and morphologic organization of AF as measured by frequency domain analysis and RQA of coronary sinus EGMs following FIRM ablation.

## 2 METHODS

### 2.1 Study population and rotor selection

**Figure 1:**
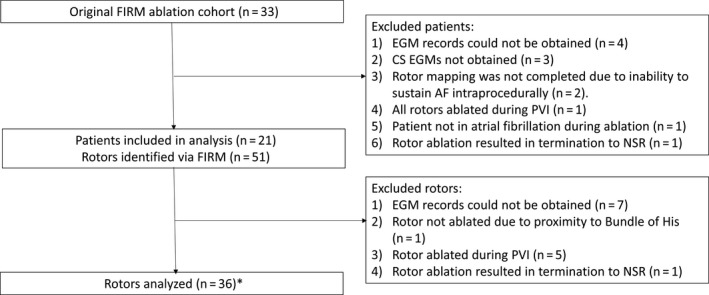
Study design flowchart. *1 rotor analyzed consisted of 2 LIPV rotors on basket catheter mapping that were ablated simultaneously

For the purpose of this study, we formed a retrospective cohort of 33 consecutive patients undergoing FIRM mapping and ablation as part of their clinical care for medically refractory AF at Duke University Hospital between October 2012 and Jun 2015 (Figure 1). For each patient, we constructed a dataset of coronary sinus bipolar EGMs immediately before and after ablation of each FIRM rotor. We limited our analysis to rotors where the cardiac rhythm immediately before and after ablation was AF, and where it was clear from the recording system when rotor ablation initiated and terminated.

Patients were excluded if intraprocedural EGM records could not be obtained (n = 4/33, 12%), if EGMs were not measured in the coronary sinus (n = 3/33, 9%), or if rotor mapping was not completed due to inability to sustain AF intraprocedurally (n = 2/33, 6%). One patient was also excluded because the patient was in an atrial tachycardia during ablation, and another patient was excluded because only one rotor was ablated, with termination to normal sinus rhythm during ablation of this rotor. Finally, one patient was excluded because all identified rotors were ablated simultaneously during pulmonary vein isolation, and thus immediate pre‐ablation and post‐ablation tracings could not be identified (n = 1/33, 3%). The final study cohort consisted of 21 patients.

Of these patients, 51 rotors were identified with FIRM mapping. Rotors were excluded from the current analysis if EGM data were unavailable due to noise or other corruption during ablation of the rotor (7/51, 14%), if the rotor was ablated simultaneously with pulmonary vein isolation (5/51, 10%), or if ablation of the rotor resulted in immediate termination of AF to sinus rhythm (n = 1/51, 2%). An additional rotor identified on the septal right atrium was not ablated due to its proximity to the Bundle of His, and thus was also excluded. In some instances, the basket catheter often overlapped tissue at the os of pulmonary vein. By convention, if the rotor was located just outside the vein os, it was assigned to that vein in the classification schema. In one patient, two rotors in close proximity near the left inferior pulmonary vein were ablated simultaneously. These two rotors were treated as one rotor in the current analysis. Consequently, a total of 36 ablation sites were analyzed, with one site consisting of simultaneous ablation of two rotors.

### 2.2 Ablation procedure

FIRM ablation procedures were performed by one of three experienced electrophysiologists at Duke University Hospital, each with greater than 4 years of experience with catheter ablation for AF. During the procedures, three‐dimensional electroanatomic mapping was performed of the right and left cardiac atria with the CARTO (Biosense Webster, Diamond Bar), EnSite NavX (St. Jude Medical, St. Paul), or Rhythmia (Boston Scientific, Nantick) mapping systems. After heparinization (activated clotting time > 300 seconds, target 350‐450), a 64‐pole basket mapping catheter (FIRMap, Abbott) was introduced into the right and left atrium (after transseptal puncture) for FIRM mapping. Simultaneous one‐minute unipolar electrograms were then recorded with the basket catheter. Using a proprietary algorithm (RhythmView, Abbott), phase mapping of the atrial activity in each atrium was then performed, and local rotors, defined as regions of sustained (≥3 cycles) circular activation patterns, were identified as targets for ablation. Radiofrequency energy was then delivered to the rotor sites until elimination of local bipolar voltage (<0.1 mV) and elimination of rotor‐like activity was confirmed with FIRM remapping. In all procedures included in this analysis, a decapolar 2‐5–2 mm spaced catheter was used and five non‐overlapping bipolar electrograms were recorded continuously throughout the case, including immediately before and after rotor site ablations. These coronary sinus EGMs were sampled at a rate of 1000 Hz, and were bandwidth filtered at the time of measurement to remove baseline voltage wander, high‐frequency noise, and notch‐filtered to remove 60 Hz frequency contamination from the alternating‐current power source. Simultaneous surface electrocardiogram recordings were also recorded during the procedure. All other aspects of the ablation procedure, including the inclusion of pulmonary vein isolation or other ablation lesions, were left to the discretion of the operator.

### 2.3 Electrogram exportation and analysis

For each rotor included in the study, a 7‐10 second digital coronary sinus EGM tracing was exported immediately prior to and after radiofrequency ablation. Exported tracings contained four surface ECG leads (I, avF, V1, V6), as well as five bipolar coronary sinus EGMs. EGM analysis was based 3 contiguous coronary sinus electrograms based upon optimal signal to noise ratios. On 3 occaisons, EGM selection was restricted to two (n = 2) and one (n = 1) bipoles.

### 2.4 Electrogram post‐processing and analysis

**Figure 2:**
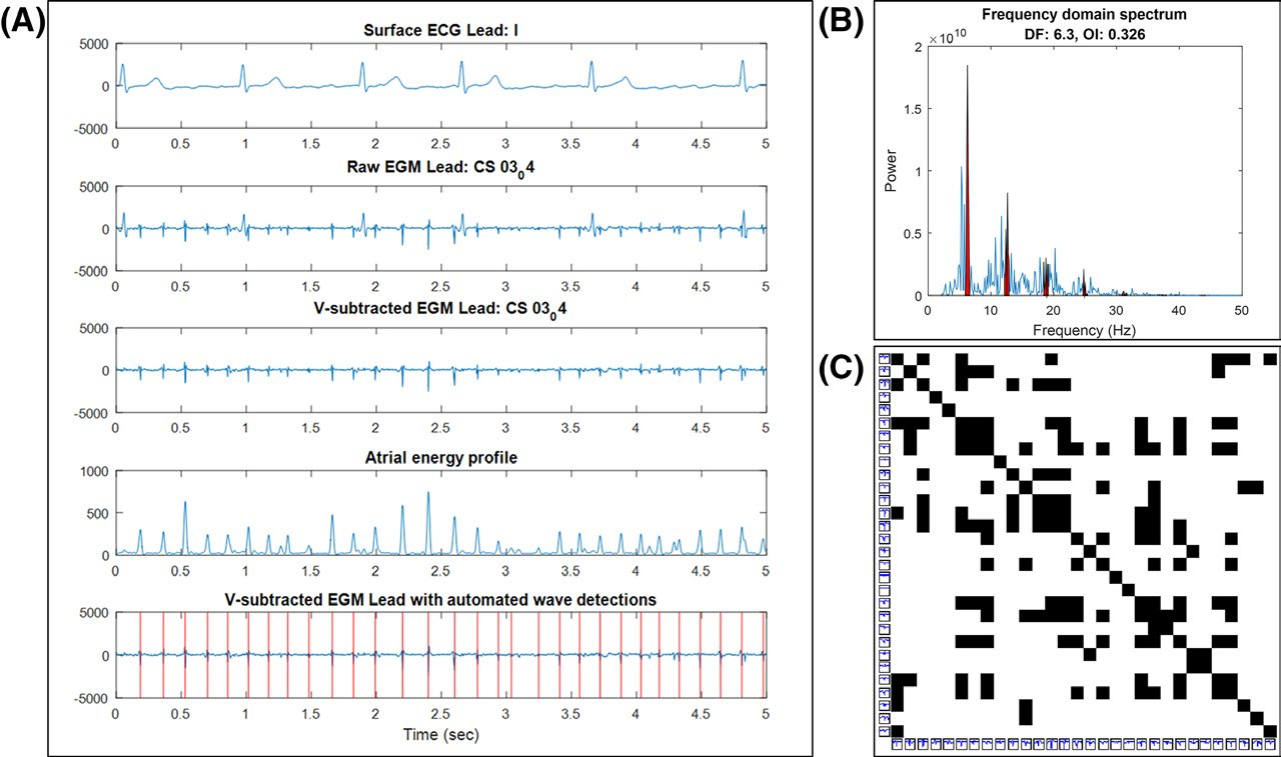
EGM postprocessing and analysis (A) An example of all EGM postprocessing steps, including ventricular‐wave subtraction, atrial energy profile generation, and automated atrial wave detection. (B) Example calculation of DF and OI from the fast Fourier transformation of the atrial energy profile in A. The red areas indicate harmonics of the DF used to calculate the OI. (C) Example waveform morphology recurrence plot from the automatic wave detections in A

All EGMs were analyzed using custom software developed in MATLAB (The MathWorks, Inc). A detailed description of the electrogram processing is presented in Appendix‐Section 1. EGM post‐processing steps included: (a) semiautomatic ventricular wave subtraction using median‐beat subtraction (Appendix‐Section 2), (b) generation of an activation energy profile by low‐pass filtering of the rectified atrial signal,^13^, ^15^ and (c) automated detection of atrial activation waves using an iterative search method^13^ (Figure 2). In the RQA analysis, morphology recurrence plots were generated from the detected atrial waves, and quantitative descriptors of the recurrence plots were computed.^13^ In the frequency domain analysis, fast Fourier transformation of the activation energy profiles were performed to compute a dominant frequency (DF) and organization index (OI).^10^, ^11^

### 2.5 Statistical analysis

Baseline patient characteristics are reported as an average ± standard deviation or median and quartiles for ordinal and continuous variables, and as number and proportion for nominal variables. To compare the change in RQA (Appendix‐Section 3) and frequency domain measures of AF organization before and after ablation of FIRM‐identified rotors, each parameter was first averaged from up to three coronary sinus EGMs. Normality of the pre‐ablation to post‐ablation difference was then assessed by taking the majority result of Kolmogorov‐Smirnov, Anderson‐Darling, and Jarque‐Bera testing with a significance level of 0.05. Differences in pre‐ and post‐ablation parameters were assessed using paired‐sample *t* testing for normally distributed parameters and Wilcoxon signed‐rank testing for non‐normally distributed parameters. To assess for the effect of non‐independence in rotors from the same patient, a sensitivity analysis using only first‐ablated rotors during the procedure was also performed. Correlations between RQA and frequency domain parameters were assessed using Pearson's correlation coefficients. Finally, contingency plots were reported to compare rotors that showed organization on RQA parameters with rotors that showed an increase in OI, and independence of distributions assessed with a chi‐squared test of independence.

The study was approved by the Duke University institutional review board.

## 3 RESULTS

### 3.1 Patient and rotor characteristics

Baseline patient characteristics and procedural data related to FIRM mapping are reported in Table 1. The mean age was 62.7 ± 11.1 years, 15 (71%) were male, and a minority had paroxysmal AF (n = 6, 29%). Of the procedures, 9 (43%) were redo AF ablation procedures. During FIRM mapping, an average of 2.43 ± 0.85 rotors were identified in each patient, with a total of 51 identified rotors. Of these rotors only one right atrial septal rotor was not ablated due to proximity to the Bundle of His. Due to the specifications required for EGM analysis, for the purpose of this analysis 36 rotor ablation sites were ultimately included in the study, consisting of 1.76 ± 0.87 per patient. The distribution of the rotors are shown in Table 2, including 16 (44%) rotors in the right atrium. Freedom from recurrent atrial tachycardia (AT) or AF > 30 seconds after the 3‐month blanking period is shown in Appendix 4. The overall AT/AF free survival was 67% at 1‐year.

**Table 1:** Patient and procedure characteristics

Patient characteristic	Total cohort, n = 21 (%)
Age (years), mean +/− SD	62.7 ± 11.1
Male	15 (71.4)
Caucasian	17 (80.9)
AF Type	
Paroxysmal	6 (28.6)
Persistent	12 (57.1)
Long‐standing persistent	3 (14.3)
CHA_2_DS_2_‐VASc, mean ± SD	2.7 ± 2.0
Heart failure	7 (33.3)
Diabetes	3 (14.3)
Hypertension	16 (76.2)
Obstructive sleep apnea	9 (42.9)
Peripheral vascular disease	3 (14.3)
Coronary artery disease	9 (42.9)
Prior stroke or transient ischemic attack	0
Previously failed > 1 antiarrhythmic drug	14 (66.6)
Beta‐blocker	10 (47.6)
Redo Ablation	9 (42.9)
LVEF (%), mean ± SD	52.8 ± 9.95
LA diameter (cm), median (IQR)	4.65 (4.4, 50)
# Rotors identified by FIRM, mean ± SD	2.43 ± 0.85
# Rotors ablated, mean ± SD	2.38 ± 0.84
# Rotors analyzed, mean ± SD	1.76 ± 0.87

**Table 2:** Anatomic distribution of rotors

Rotor location	Rotor count
**Right Atrium**	**16**
Lateral RA/Crista	7
Medial/Septal RA	5
Posterior RA	4
**Left Atrium**	**20**
Anterior LA	2
Roof	5
LAA	3
LSPV or LIPV	4
Posterior/ Inferior Wall	5
Mitral Isthmus	1
**Total:**	**36**

Abbreviations: LA, left atrium; LAA, left atrial appendage; LIPV, left inferior pulmonary vein; LSPV, left superior pulmonary vein; RA, right atrium.

### 3.2 Changes in measures of AF organization following FIRM ablation

**Figure 3:**
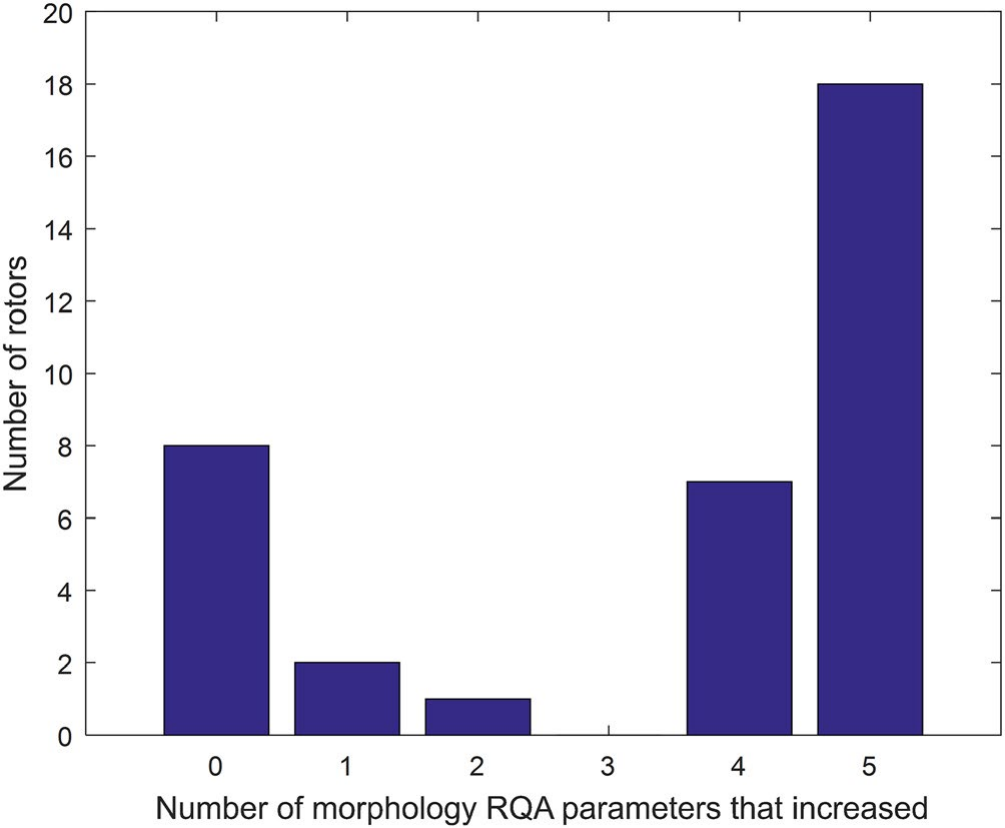
Histogram of rotors by the number of morphology RQA parameters that increased

Results of morphology RQA and frequency domain analysis of the coronary sinus EGMs are summarized in Table 3. Of all parameters, only changes in diagonal line length were found to be normally distributed; because parametric and nonparametric testing were concordant for this parameter, nonparametric results are reported for all parameters. All post‐ablation RQA parameters exhibited an increase compared to pre‐ablation. Recurrence rate (RR) had a median increase of 2.2% (*P* = .03), determinism (DET) had a median increased of 5.6% (*P* = .005), diagonal line length (L) had a median increase of 0.04 (*P* = .03), laminarity (LAM) had a median increase of 4.0% (p‐0.005), and trapping time (TT) had a median increase of 0.073 (*P* = .01). Of the rotors studied, 18/36 rotors (50%) showed an increase in all morphology RQA parameters, and 25/36 rotors (69%) showed an increase in at least 4 of the 5 RQA parameters (Figure 3). Additionally, 26/36 rotors (72%) demonstrated an increase in organizational index (OI). The median increase in the OI was 2.2% (*P* = .01). In total, 14/36 (39%) rotors showed organizational changes in all morphology parameters and OI, and 19/36 (53%) rotors showed organizational changes in 4 of 5 morphology parameters and OI (Table 3). In order to ensure that organization results were not due to progressive ablation of rotors, we conducted a sensitivity analysis limited to ablation of first rotors only. The significance of pre‐ablation versus post‐ablation differences in morphology RQA parameters and OI were strengthened in subgroup analysis of only first‐ablated rotors (Appendix 5).

**Table 3:** Change in atrial fibrillation organization with FIRM ablation

EGM parameter	Pre‐ablation value	Post‐ablation value	Difference	*P*‐value
Morph RR	0.116 (0.0517, 0.203)	0.155 (0.0734, 0.207)	0.0216 (−0.0163, 0.0568)	.0322
Morph DET	0.244 (0.123, 0.337)	0.277 (0.159, 0.441)	0.0556 (−0.0205, 0.134)	.00474
Morph L*	1.17 (1.07, 1.36)	1.22 (1.11, 1.45)	0.04 (−0.0483, 0.21)	.0331
	1.25 ± 0.26	1.33 ± 0.29	0.076 ± 0.223	.0228*
Morph LAM	0.332 (0.212, 0.445)	0.421 (0.257, 0.543)	0.074 (−0.0182, 0.138)	.00523
Morph TT	1.3 (1.17, 1.67)	1.42 (1.19, 1.8)	0.0733 (−0.0417, 0.285)	.00886
DF (Hz)	5.49 (4.84, 6.17)	5.64 (4.91, 6.21)	‐0.0875 (−0.298, 0.225)	.635
OI	0.333 (0.264, 0.375)	0.333 (0.275, 0.414)	0.0221 (−0.0052, 0.0738)	.0097

All values reported as medians (quartiles). *P*‐values calculated using one‐tailed Wilcoxon signed‐rank testing unless otherwise specified.

Abbreviations: DET, determinism; DF, dominant frequency; EGM, (intracardiac) electrogram; L, diagonal line length; LAM, laminarity; morph, morphology; OI, organizational index; RR, recurrence rate; TT, trapping time.

* Due to discordant results on normality testing, nonparametric (top) and one‐tailed paired t‐test of mean differences (bottom) results reported.

We also conducted a sensitivity analysis comparing changes in morphology RQA and OI parameters after FIRM ablation of right atrial versus left atrial rotors. While there was overlap in the interquartile ranges, the changes in RQA and OI parameters were numerically higher after RA rotor ablation (Appendix 6).

Overall, there was no significant change in the dominant frequency (DF, *P* = .635). In subgroup analysis of the 14 rotors which showed organization in all RQA parameters and OI, as well as subgroup analysis of the 19 rotors which showed organization in at least 4 out of 5 morphology parameters in addition to OI, there was no evidence of significant change in the DF (median change in DF and quartiles −0.055 [−0.41, 0.23], *P* = .35 for the 14 rotors and 0.033 [−0.16, 0.24], *P* = .62 for the 19 rotors).

### 3.3 Association between OI and morphology RQA parameters

**Figure 4:**
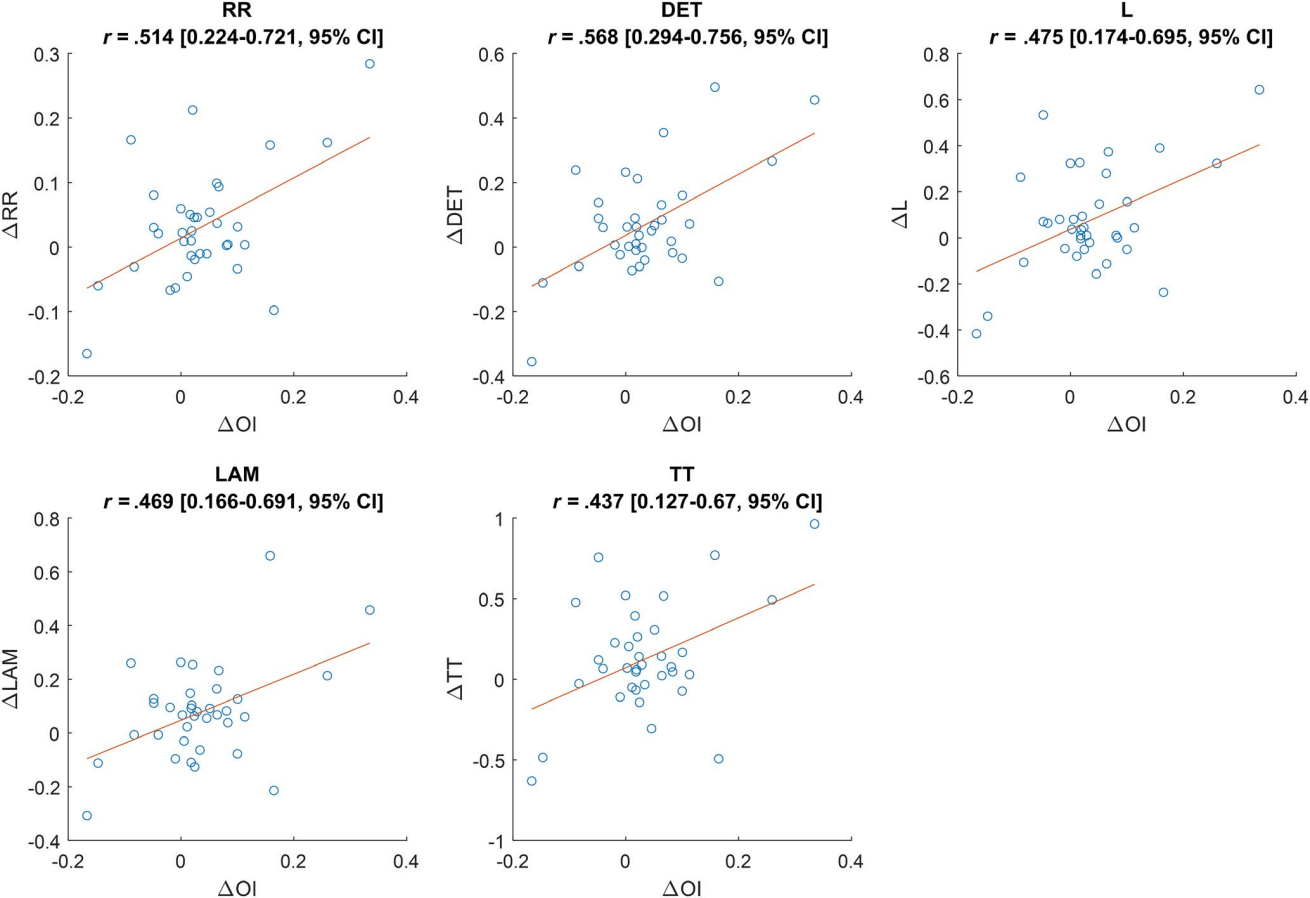
Scatter plots and Pearson correlation coefficients between morphology RQA parameters and organizational index for all rotors. All correlations significant (*P* < .01)

All morphology parameters showed a moderate, positive correlation with OI (Figure 4). However, there was no association observed between the number of morphology parameters that increased in a particular rotor, and whether that rotor also exhibited an increase in OI (*P* = .446, Table 4).

**Table 4:** Comparison of morphology and frequency domain changes during FIRM ablation*

	Morph0	Morph1+	Morph2+	Morph3+	Morph4+	Morph5+	Total
OI−	4	0	0	0	2	4	10
OI+	4	2	1	0	5	14	26
Total	8	2	1	0	7	18	36

* Contingency table comparing rotors that showed an increase in OI with rotors that showed an increase in morphology RQA parameters. Rotors that showed in increase in OI did not show an association with rotors that showed changes in morphology (*χ* ^2^ = 3.40, *P* = .446).

## 4 DISCUSSION

While the importance of spiral rotors in sustaining AF has long been hypothesized, the effects of rotor ablation on AF conduction patterns have not been well‐characterized. Efforts to categorize patients that show AF slowing, organization, or termination have been challenged with limited reproducibility, and the significance of these findings during FIRM ablation are unclear. By comparing quantitative measures of atrial EGM properties before and after FIRM rotor ablation, the current study provides several findings regarding FIRM rotor ablation. First, we present strong evidence that FIRM ablation of rotors and focal sources causes measurable changes to both the waveform morphology and activation patterns in the coronary sinus that suggest a more organized fibrillary activation in the left atrium. Second, by comparing the behavior of OI with morphology RQA in these rotors, we find that while changes in these parameters are correlated, they also likely contribute independent information on AF organization. Finally, we find no evidence of fibrillary rate slowing based on our analysis of the coronary sinus DF, even in subgroup analysis, suggesting that FIRM rotor ablation may lead to organization of AF even in the absence of the fibrillary rate slowing that is commonly reported.

While there have been several applications of frequency domain analysis and morphology RQA techniques in the setting of catheter ablation of AF, to the best of our knowledge, there has been no study to date that has shown changes in EGMs as a result of directed rotor ablation (outside of conventional pulmonary vein isolation). Previous studies have suggested the role of DF,^19^ OI,^11^ and RQA^14^ in the prediction of short‐ and long‐term outcomes in AF ablation. Several other studies have also suggested that both OI^10^ and morphology RQA^13^, ^20^ may describe foci of atrial organization critical to the maintenance of AF. Moreover Jarman et al have observed that during procedures where pulmonary vein isolation (PVI) lines are in close proximity to areas of high OI, there is a resultant increase in OI at sites distant from the PVI line.^10^ The current study adds to these findings in two distinct ways. First, our finding that FIRM rotor ablation causes an increase in coronary sinus OI are consistent with the findings of Jarman et al,^10^ and suggest that ablation of sites with high OI may have similar effects relative to rotor ablation. Second, we found that rotor ablation leads to changes in EGM waveform stability as well, providing further evidence that atrial substrate ablation can modify the global fibrillatory organization.

Our results also suggest that organization can be seen, even without changes in the DF. Prior work from other groups has shown this as well.^21^ Our results also confirm and extend prior work demonstrating that OI is a more sensitive fast Fourier transform marker of AF organization.^10^ The lack of changes in the DF may be because the DF of AF at a site distant from the ablated rotors are affected by other fibrillatory properties of the global atrial substrate.

Given that changes to both OI and morphology RQA occur directly after rotor ablation, it is tempting to assume that they are changing via the same mechanism. Indeed, we found that all morphology RQA parameters were correlated with OI, suggesting that they were both related to rotor ablation. However, morphology RQA and OI measure distinctly different properties of the EGM. Just as morphology RQA measures solely waveform stability and is blinded to activation cycle lengths, OI as defined in this study measures solely the temporal patterns of the atrial energy profile and is blinded to waveform morphologies. This may be why the rotors which exhibited a change in morphology RQA were not associated with the rotors which exhibited a change in OI. This result is consistent with findings by Ng et al^13^ which also showed independence of morphology RQA and frequency domain parameters. Together, these findings suggest that waveform morphology analysis and activation pattern analysis may impart additional information in quantifying changes in the properties of the underlying AF.

Results following FIRM ablation have been highly varied with some centers reporting no apparent improvement in freedom from AF with adjunctive FIRM ablation. Assuming that focal sources of AF are important in the initiation and maintenance of AF, there are several potential explanations for the wide variation in results following FIRM. First, it is possible that the current tools used do not adequately and consistently cover or map the entire atrial myocardium. A second possibility is that the mapping is adequate, but that the ablation is ineffective, possibly because it is not targeted to an appropriate intraprocedural endpoint. Elimination of apparent rotors on phase mapping or elimination of local atrial voltage may be informative yet not completely sufficient to guide rotor ablation. Future studies of atrial activation before and after rotor ablation should investigate the relationship between frequency domain organization (OI), morphologic organization, and post‐procedural outcomes (ie, long‐term freedom from AF).

### 4.1 Limitations

There are several important limitations to consider when assessing the results from these analyses. First, it is important to note that the assessment of left atrial activation was based on a convenience sample from the coronary sinus catheter (as is the case in most static assessments of left atrial arrhythmia). Analysis of coronary sinus electrograms may have been more useful in appreciating the changes imparted by left atrial ablation. The lack of a sample of right atrial electrograms is a limitation of our study design*.* Second, because all rotors were targeted and there were no controls, it is possible that the change in organization was due to debulking a small amount of atrial tissue rather than a direct effect of rotor ablation. We only utilized FIRM ablation. Therefore, these findings might not apply to rotor ablation using other methods such as noninvasive ECG mapping. Finally, at present, there is no gold standard for rotor detection (endocardial basket mapping, epicardial mapping, body surface activation mapping, etc) and the "rotors" that were ablated without subsequent organization may not have been rotors at all. A potentially controversial interpretation is that perhaps this was stable passive rotational activity rather than rotational activity due to an AF driver. Several of these limitations may be clarified in the future as our understanding of rotor behavior evolves further.

## 5 CONCLUSION

This study demonstrates that FIRM ablation of rotors and focal sources causes measurable changes to both the waveform morphology and activation patterns in the coronary sinus that suggest a more regularized fibrillatory activation in the left atrium. Moreover RQA morphology analysis and OI provide correlated but independent information and may impart crucial information regarding underlying AF organization and may be useful in quantifying the acute response to rotor ablation as well as providing intraprocedural guidance to ablation of focal sources.

## DISCLOSURES

PRL, DJF, ASB, JPD, and KPJ have no relevant disclosures. JPP receives grants from Abbott, American Heart Association, Association for the Advancement of Medical Instrumentation, Bayer, Boston Scientific, and Philips and serves as a consultant to Abbott, Allergan, ARCA Biopharma, Biotronik, Boston Scientific, LivaNova, Medtronic, Milestone, Sanofi, Philips, and Up‐to‐Date.
