# Supplementary material for: Focal impulse and rotor modulation of atrial rotors during atrial fibrillation leads to organization of left atrial activation as reflected by waveform morphology recurrence quantification analysis and organizational index
Source: J Arrhythm. 2020 Feb 24;36(2):311–8. doi: 10.1002/joa3.12311 (PMC7132180; doi:10.1002/joa3.12311)
Supplement: Supplementary file 1 [file JOA3-36-311-s001.docx]

**Appendix – Section 1: Electrogram Post-Processing and Analysis**

*Ventricular Wave Detection and Subtraction*

Semi-automated ventricular wave subtraction of each CS EGM was performed by first detecting QRS complexes on the surface ECGs to identify the time-points at which ventricular activity occurred. Briefly, QRS detection was achieved by generating a composite root-mean-squared tracing by summing the squared voltages of all individual ECG leads (I, aVF, V1, V6), smoothing with a moving average filter, and solving for the inflection point of the negative slope of the composite ECG tracing. To optimize QRS detection results, 160ms windows were taken around each detection and averaged to generate an archetypal QRS wave, and this wave was compared to the composite ECG with a correlation function. All maxima of the correlation function greater than 33% of the maximal correlation were considered QRS detections.

Semi-automated ventricular wave subtraction of each CS EGM lead was performed after QRS detection using an automated median-beat subtraction method, followed by visual inspection and manual correction of the ventricular wave subtractions using a graphical user interface developed in MATLAB. In the automated median-beat subtraction step, an archetypal ventricular wave was generated by first taking a 110ms window of the EGM tracing around each QRS detection (starting 40ms before the detection and ending 70ms after), and the median of the ensemble of these EGM windows was computed to generate an archetypal ventricular wave on that EGM lead. This archetypal waveform was then fit to the EGM at each QRS detection site using a least squares method, with three degrees of freedom allowed for proportional scaling of the archetypal wave, vertical translation to account for possible local voltage changes related to underlying atrial activity, and horizontal translation up to 10ms to account for cardiac motion causing changes in QRS wave to EGM ventricular wave latency. The fitted waveform was then subtracted. After automated ventricular wave subtraction, all EGM tracings were manually inspected. In tracings with no obvious ventricular activity, the raw EGM tracings were used for subsequent analyses. In tracings where residual ventricular activity or poor ventricular wave fitting was noted, manual corrections to the original ventricular wave fitting were performed using a custom graphical user interface (**Appendix – Section 2**).

*Atrial energy profile generation*

After generation of ventricular wave-subtracted EGMs, a time profile characterizing the energy of the underlying atrial activity was computed using methods similar to Ng et al.^1^ This atrial energy profile was computed by high-pass filtering the ventricular wave-subtracted EGM with a cutoff frequency of 40Hz, rectification of all negative voltages of the resulting signal, then low-pass filtering the rectified signal at a cutoff of 30Hz. These energy profiles were subsequently utilized in atrial activation detection and frequency domain analysis steps.

*Atrial activation detection*

Atrial activation detection was also performed in a manner similar to Ng et al.^1^ First, initial detections were generated iteratively by finding all local maxima of the atrial energy profile. In each iteration during the first step, the next highest energy peak was selected as a detection. After each detection, a blanking window of 40ms was created around the detection before the next iteration, and candidate energy peaks were removed from further consideration inside the window. During each iteration, mean and median CL were calculated. Stop criteria were met when the mean CL was lower than a prespecified upper bound CL (275ms), and either the mean CL was less than median CL plus 5ms, or the magnitude of the last energy peak was 20% the magnitude of the previous energy peak. The prespecified upper bound was changed to 150ms for one rotor with dominant frequencies greater than 7Hz where first attempts with an upper bound of 275ms generated poor detection results on visual inspection. After initial detections were generated, intervals between detections greater than 1.5 times the median cycle length (CL) were checked for atrial activity to increase the sensitivity of the algorithm to possibly missed detections.

*Recurrence Plot Generation*

From the automated atrial wave detections, recurrence plots (RP) describing EGM morphology were generated in a manner similar to Gordon et al.^2^ First, a set of atrial waveforms that was generated from ventricular wave-subtracted EGMs with atrial wave detections. Each waveform was generated by taking a 70ms window around the atrial energy peak used to detect the atrial wave. Second, each pair of waveforms was compared by calculating a normalized cross-correlation function. A maximal cross-correlation coefficient greater than 0.8 was considered to be a match.

*Recurrence quantification analysis*

Quantitative descriptors of the generated morphology RPs were calculated based on commonly-utilized measures^3^. These measures characterize the density of the recurrence plot (recurrence rate – RR), as well as the characteristics of diagonal lines (determinism – DET, and diagonal line length – L) and vertical lines (laminarity – LAM, and trapping time – TT). A list of the measures and their definitions can be found in **Appendix – Section 3**.

*Frequency domain electrogram analysis*

Using widely-used methods^4-6^, frequency domain analysis was performed by taking the fast Fourier transformation of the atrial energy profile generated during EGM post-processing. The spectrum was only computed between 2Hz and 50Hz frequencies. The DF was defined as the frequency of the highest spectral power, and the organizational index (OI) was computed as a ratio of the power of the spectrum at the DF and all higher-order harmonics divided by the power of the total spectrum between 2Hz and 50Hz. All spectral power within 0.375Hz of the DF or DF harmonics contributed to the OI (**Figure 2B**).

**Appendix – Section 2**: **Screenshot of the GUI used in semi-automated ventricular wave subtraction**


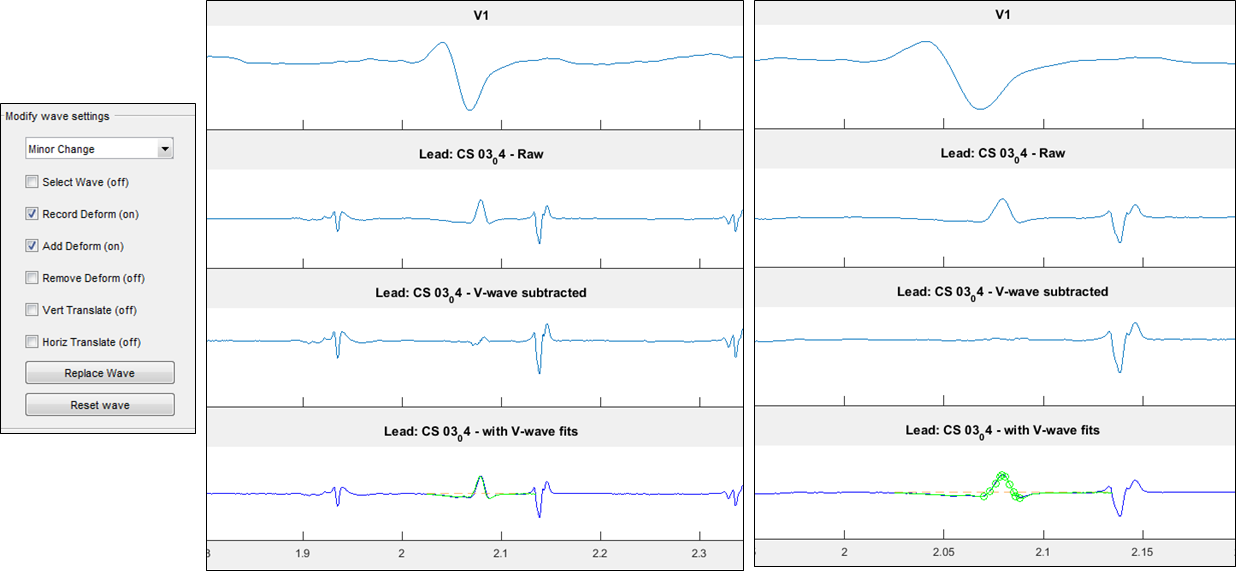


Screenshots of graphical user interface before (left tracings) and after (right tracings) manual correction of ventricular wave fittings. Residual ventricular activity (left tracings, center of third panel) is removed after manual correction (right tracings, center of third panel). On bottom panels, the blue line represents the raw exported CS EGMs. The green line represents the fitted ventricular wave for subtraction, with green circles on the right bottom panel representing manual changes to the fitted wave. The orange dotted line represents the baseline of the archetypal ventricular wave, assumed to be zero voltage.

**Appendix – Section 3: Definition of RQA parameters**

**Recurrence rate (RR)** – the proportion of pairwise comparisons (excluding self-comparisons) that were determined to be recurrences:

$RR= \frac{1}{N(N+1)}\sum_{i=1}^{N} \sum_{j=1}^{i-1} R\left( i,j \right)$ (Eq. 3).

**Determinism (DET)** – the proportion of recurrences that were part of a diagonal line:

$DET= \frac{\sum_{l=l_{min}}^{N} \left. l*n(l \right)}{\sum_{i,j=1}^{N} \left. R(i,j \right)}$ (Eq. 4),

where *l* is the diagonal line length*, n(l)* is the number of diagonal lines that are of length *l*, and *l_min_* is the prespecified minimum diagonal line length (set equal to 2 for the current analysis). For the current analysis, recurrences from self-comparisons were not counted in this measure.

**Diagonal line length (L)** – the average diagonal line length of the recurrence plot:

$L= \frac{\sum_{l=1}^{N} \left. l*n(l \right)}{\sum_{l=1}^{N} \left. n(l \right)}$ (Eq. 5),

where again *l* is the diagonal line length*, n(l)* is the number of diagonal lines that are of length *l*. Here again, self-comparisons were not counted.

**Laminarity (LAM)** – the proportion of recurrences that were part of a vertical line:

LAM$= \frac{\sum_{v=v_{min}}^{N} \left. v*n(v \right)}{\sum_{i,j=1}^{N} \left. R(i,j \right)}$ (Eq. 6),

where *v* is the vertical line length and *n(v)* is the number of vertical lines of length *v*, and *v_min_* is the prespecified minimum vertical line (set equal to 2 for the current analysis).

**Trapping time (TT)** – the average vertical line length:

$TT= \frac{\sum_{l=1}^{N} \left. l*n(l \right)}{\sum_{l=1}^{N} \left. n(l \right)}$ (Eq. 7).

**Appendix - Section 4**: Freedom from recurrent atrial tachycardia (AT) or AF >30 seconds after the 3 month blanking period is shown in the Kaplan-Maier figure below with accompanying 95% confidence intervals. The overall AT/AF free survival was 67% at 1-year.


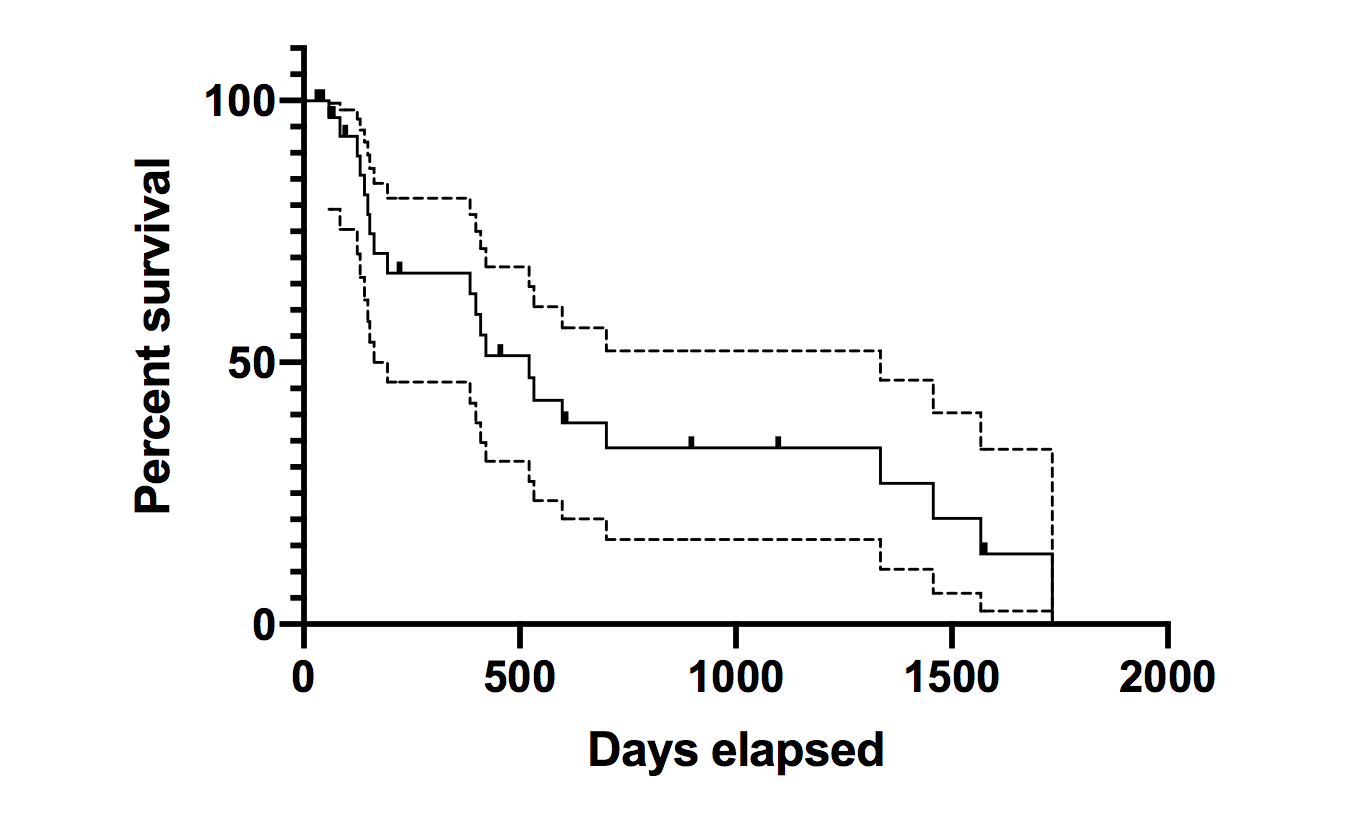


**Appendix – Section 5: Rotor Subgroup Analysis – Changes in AF Organization during Ablation of First Rotors Identified via FIRM**

| EGM parameter | Pre-ablation value | Post-ablation value | Difference | *p-value* |
| --- | --- | --- | --- | --- |
| Morph RR | 0.110 (0.0391, 0.176) | 0.145 (0.0654, 0.254) | 0.0301 (0.00328, 0.0698) | 0.0021 |
| Morph DET | 0.169 (0.113, 0.321) | 0.283 (0.14, 0.401) | 0.066 (0.011, 0.151) | 0.00043 |
| Morph L | 1.13 (1.06, 1.27) | 1.19 (1.1, 1.53) | 0.04 (0.0075, 0.265) | 0.0083 |
| Morph LAM | 0.309 (0.192, 0.398) | 0.421 (0.245, 0.533) | 0.082 (0.0343, 0.176) | 0.00043 |
| Morph TT | 1.25 (1.14, 1.48) | 1.42 (1.18, 1.87) | 0.07 (0.0275, 0.325) | 0.00071 |
| OI | 0.318 (0.26, 0.349) | 0.325 (0.269, 0.389) | 0.021 (0.00225, 0.0815) | 0.0164 |
|  |  |  |  |  |
|  |  |  |  |  |

**Table A5-1: Changes in AF Organization on Subgroup of all First-Ablated Rotors.** All values reported as medians (quartiles). P-values were calculated using one-tailed Wilcoxon signed-rank testing. Abbreviations: EGM, (intracardiac) electrogram; morph, morphology; RR, recurrence rate; DET, determinism; L, diagonal line length; LAM, laminarity; TT, trapping time; OI, organizational index.

| Rotor Location | Rotor Count |
| --- | --- |
| **Right Atrium** | **11** |
| Lateral RA/Crista | 5 |
| Medial/Septal RA | 2 |
| Posterior RA | 4 |
|  |  |
| **Left Atrium** | **10** |
| Anterior LA | 0 |
| Roof | 2 |
| LAA | 2 |
| LSPV or LIPV | 2 |
| Posterior/ Inferior Wall | 3 |
| Mitral Isthmus | 1 |
|  |  |
| **Total:** | **21** |

**Table A5-2:** Anatomic distribution rotors in the first-ablated rotors subgroup

**Appendix - Section 6**: Changes in morphology RQA and organization index according to location of rotors in the right atrium versus left atrium.

**
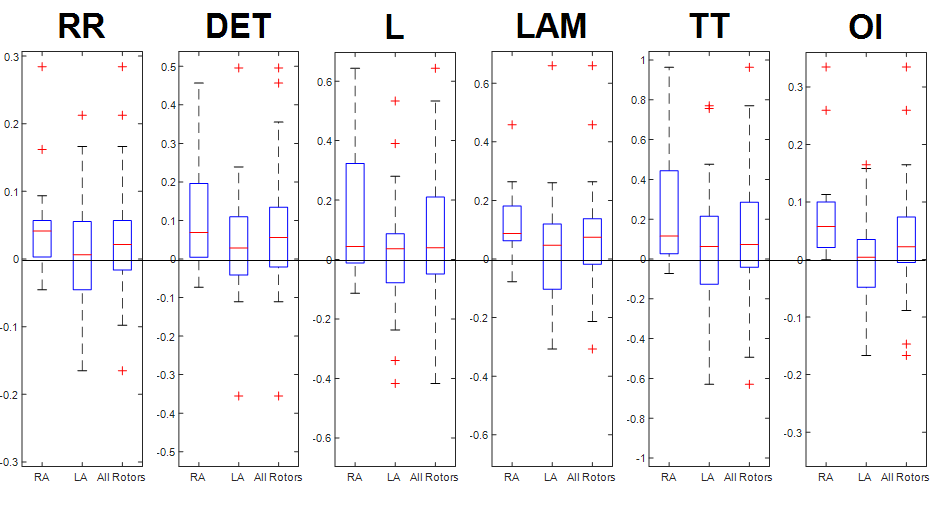
**

Boxplots of the changes in morphology RQA and OI parameters during FIRM ablation. Red line indicates median changes, boxes indicate the 25^th^ and 75^th^ percentiles, and dotted lines indicate most extreme values not including outliers*.*

**References**

1. Ng J, Sehgal V, Ng JK, Gordon D and Goldberger JJ. Iterative method to detect atrial activations and measure cycle length from electrograms during atrial fibrillation. *IEEE Trans Biomed Eng*. 2014;61:273-8.

2. Gordon D, Goldberger JJ, Arora R, Aistrup GL and Ng J. Searching for "order" in atrial fibrillation using electrogram morphology recurrence plots. *Comput Biol Med*. 2015;65:220-8.

3. Marwan N, Carmen Romano M, Thiel M and Kurths J. Recurrence plots for the analysis of complex systems. *Physics Reports*. 2007;438:237-329.

4. Everett Iv TH, Akar JG, Kok L-C, Moorman JR and Haines DE. Use of global atrial fibrillation organization to optimize the success of burst pace termination. *Journal of the American College of Cardiology*. 2002;40:1831-1840.

5. Jarman JWE, Wong T, Kojodjojo P, Spohr H, Davies JER, Roughton M, Francis DP, Kanagaratnam P, O'Neill MD, Markides V, Davies DW and Peters NS. Organizational Index Mapping to Identify Focal Sources During Persistent Atrial Fibrillation. *Journal of Cardiovascular Electrophysiology*. 2014;25:355-363 9p.

6. Jones AR, Krummen DE and Narayan SM. Non-invasive identification of stable rotors and focal sources for human atrial fibrillation: mechanistic classification of atrial fibrillation from the electrocardiogram. *Europace*. 2013;15:1249-1258.
